# Interspecific Genetic Differences and Historical Demography in South American Arowanas (Osteoglossiformes, Osteoglossidae, *Osteoglossum*)

**DOI:** 10.3390/genes10090693

**Published:** 2019-09-09

**Authors:** Fernando Henrique Santos de Souza, Manolo Fernandez Perez, Luiz Antônio Carlos Bertollo, Ezequiel Aguiar de Oliveira, Sebastien Lavoué, Carla Cristina Gestich, Petr Ráb, Tariq Ezaz, Thomas Liehr, Patrik Ferreira Viana, Eliana Feldberg, Marcelo de Bello Cioffi

**Affiliations:** 1 Departamento de Genética e Evolução, Universidade Federal de São Carlos (UFSCar), Rodovia Washington Luiz Km. 235, C.P. 676, São Carlos, SP 13565-905, Brazil; 2 Secretaria de Estado de Educação de Mato Grosso—SEDUC-MT, Cuiabá, MT 78049-909, Brazil; 3 School of Biological Sciences, Universiti Sains Malaysia, Penang 11800, Malaysia; 4 Laboratory of Fish Genetics, Institute of Animal Physiology and Genetics, Czech Academy of Sciences, Rumburská 89, 277 21 Liběchov, Czech Republic; 5 Institute for Applied Ecology, University of Canberra, Canberra, ACT 2617, Australia; 6 Institute of Human Genetics, University Hospital Jena, 07740 Jena, Germany; 7 Instituto Nacional de Pesquisas da Amazônia, Coordenação de Biodiversidade, Laboratório de Genética Animal, Av. André Araújo 2936, Petrópolis, CEP 69067-375, Brazil

**Keywords:** fishes, cytogenetics, DArTseq, population structure, colonization pathway, genomics

## Abstract

The South American arowanas (Osteoglossiformes, Osteoglossidae, *Osteoglossum*) are emblematic species widely distributed in the Amazon and surrounding basins. Arowana species are under strong anthropogenic pressure as they are extensively exploited for ornamental and food purposes. Until now, limited genetic and cytogenetic information has been available, with only a few studies reporting to their genetic diversity and population structure. In the present study, cytogenetic and DArTseq-derived single nucleotide polymorphism (SNP) data were used to investigate the genetic diversity of the two *Osteoglossum* species, the silver arowana *O. bicirrhosum*, and the black arowana *O. ferreirai*. Both species differ in their 2n (with 2n = 54 and 56 for *O. ferreirai* and *O. bicirrhosum*, respectively) and in the composition and distribution of their repetitive DNA content, consistent with their taxonomic status as different species. Our genetic dataset was coupled with contemporary and paleogeographic niche modeling, to develop concurrent demographic models that were tested against each other with a deep learning approach in *O. bicirrhosum*. Our genetic results reveal that *O. bicirrhosum* colonized the Tocantins-Araguaia basin from the Amazon basin about one million years ago. In addition, we highlighted a higher genetic diversity of *O. bicirrhosum* in the Amazon populations in comparison to those from the Tocantins-Araguaia basin.

## 1. Introduction

Amazonia is the ecosystem hosting the highest amount of biodiversity in the world [1]. Such an extreme biodiversity level is associated with both high speciation and low extinction rates in this region [2]. South America experienced a complex geological and climatic history marked by several intense events that affected the biota in the Amazon basin (AMb), such as important and recurrent marine incursions and river course alterations [3]. The predominant causes and timing of diversification in the Neotropical region are still under examination and contra posed biogeographical hypotheses have been recently presented. These hypotheses suggest the importance of either tectonic events that occurred primarily during the Neogene [4] or more recent events that took place during Pleistocene [5]. Though there is no consensus, it is likely that events in both periods contributed to the diversification of Neotropical, including Amazonian, biota [6,7].

The ichthyofauna of the Amazon basin is the richest worldwide with representatives of many taxonomic groups [2,8]. One of its most emblematic representatives, the arowanas of the genus *Osteoglossum* (Osteoglossidae), are widely distributed in the AMb, as well as in some of its neighboring basins [9]. Two arowana species are currently recognized: the black arowana, *Osteoglossum ferreirai* and the silver arowana, *Osteoglossum bicirrhosum*. However, cryptic diversity is potentially present in the genus, as suggested by substantial differences among populations of both species, particularly at reproductive strategies and growth patterns [10,11], and morphometric characteristics [12]. *Osteoglossum ferreirai* occurs in Negro and Orinoco river basins, living mainly in acidic blackwaters, like in the Negro, Bita, and Tomo rivers [13,14]. Meanwhile, *O. bicirrhosum* lives in clean and alkaline waters of the Amazon, Tocantins-Araguaia (TAb), and Essequibo basins [9]. It is presumed that the water types act as a dispersal barrier for the species’ distributions, although there are reports of syntopy in the Demeni River, where both clean and acid waters occur [9]. Both arowana species can grow up to 1.2 meters in length and are omnivorous, feeding mainly on insects, crustaceans, mollusks, and other fishes. They have low fecundity and are mouthbrooding similar to some cichlid species, and attain sexual maturity at about two years and after spawning, males carry offspring in their mouth for about four to six weeks [9].

Both species are under anthropogenic pressures, where the silver arowana is highly exploited as a food source and the black arowana used as ornamental purposes [15,16,17,18]. The International Union for Conservation of Nature (IUCN) Red List contains only *O. ferreirai*, assigned with a LC (least concern) status, but suggest that this classification needs updating [19]. *Osteoglossum bicirrhosum* is only included in the Red Book of the Colombian freshwater fishes [20], classified as V (vulnerable), while *O. ferreirai* is considered as an En (endangered) species. Because of the significant strength of anthropogenic threat affecting these two species, and the few genetic studies available [21,22], comprehensive genetic studies are needed to propose efficient conservation strategies.

The advent of new technologies involving high-throughput sequencing to obtain large genetic datasets in non-model organisms allows investigating genetic variation from markers scattered along the species’ genome, resulting in more accurate inferences [23]. Several methods are available to develop hundreds to thousands of markers using genotyping by sequencing technologies which have been applied to infer population genetic structure and genetic diversity across multiple non-model species [24,25]. Among such strategies, DArTseq (diversity arrays technology) enriches for hypomethylated genomic regions, that are putatively under selection, with subsequent sequencing of thousands of single nucleotide polymorphisms (SNPs) [26,27].

The recent combined use of cytogenetic and refined molecular studies has allowed a better understanding of both intra- and interspecific cytogenomic organization and differentiation [28]. Recently, besides conventional cytogenetic methods, the use of comparative genomic hybridization (CGH) has been successfully applied for the genome comparisons among related species and/or among populations [29,30,31,32].

In this study, we characterized the cytogenetic and genomic patterns of natural populations from both black and silver arowanas to evaluate the genetic differentiation between and within species and to contribute to better strategies for their conservation. The genomic information was coupled with geographic climatic models to design possible demographic events that led to the current distribution of the species. For this purpose, we assessed data from several parts of the specimens’ genome isolated with DArTseq, and also retrieved information about conserved genomic regions as revealed by cytogenetics techniques, which provides a wider overview of their genetic organization.

## 2. Materials and Methods

### 2.1. Individuals Sampling

**Figure 1:**
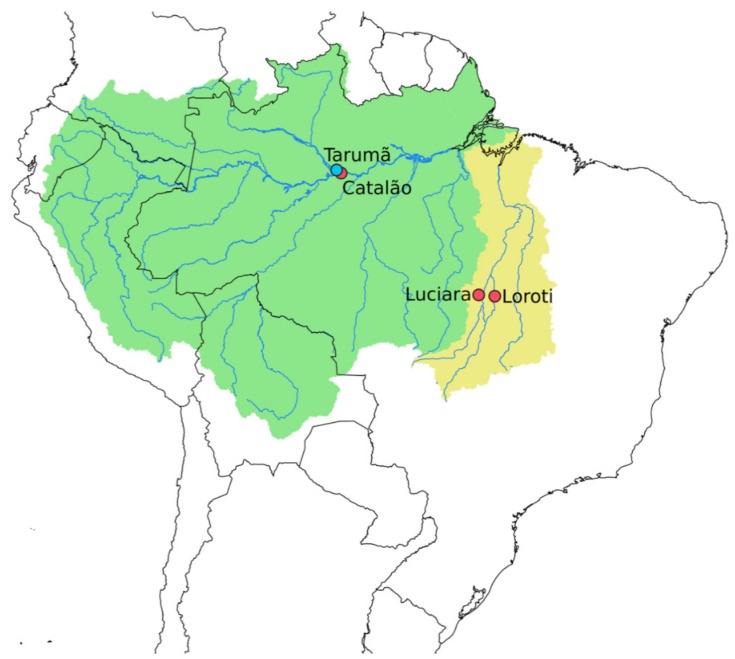
Map of Brazil and neighboring countries showing sampling sites for *Osteoglossum bicirrhossum* (red dots) and *O. ferreirai* (blue dot) from Amazon (green) and Tocantins-Araguaia (yellow) river basins.

The number, sampling sites and sex of the individuals are presented in Table 1 and Figure 1. Sample sizes for the SNP dataset are smaller than those used for the cytogenetic analyses, as some samples were obtained after the library preparation and sequencing experiment. The samples were collected following authorization of the Brazilian Environmental Agency, Instituto Chico Mendes de Conservação da Biodiversidade/ Sistema de Autorização e Informação em Biodiversidade (ICMBio/ SISBIO) (License No. 48290-1) and Sistema Nacional de Gestão do Patrimônio Genético e do Conhecimento Tradicional Associado (SISGEN, A96FF09). All specimens were identified by morphological criteria, and the voucher specimens were deposited under numbers 121638 until 121640 in the Museum of Zoology of the University of São Paulo (MZUSP).

### 2.2. Mitotic Chromosomal Preparations and Comparative Genomic Hybridization (CGH)

Mitotic chromosomes were obtained following the protocol by Bertollo et al. [33]. The experiments followed ethical and anesthesia conducts, according to the Ethics Committee on Animal Experimentation of the Universidade Federal de São Carlos (Process number CEUA 1853260315). The total genomic DNAs (gDNAs) were extracted from liver tissue by the standard phenol–chloroform–isoamyl alcohol method [34].

Two different experimental designs of comparative genomic hybridization (CGH) were used. The first one focused on interspecific genomic comparisons and, in this case, gDNAs of *O. ferreirai* and *O. bicirrhosum* from the AMb localities were labeled with Aminoallyl-dUTP - ATTO-550 (red) and Aminoallyl-dUTP - ATTO-488 (green) using Nick-translation labeling kit (Jena Bioscience, Jena, Germany), respectively, and hybridized against the chromosomal background of *O. ferreirai*. The final hybridization mixture for each slide contained 500 ng of each labeled gDNA and 25 μg of unlabeled C0t-1 DNA from both species (to block the shared repetitive sequences prepared according to Zwick et al. [35]), dissolved in 20 μL of the hybridization buffer (50% formamide, 2 × SSC, 10% SDS, 10% dextran sulfate and Denhardt’s buffer, pH 7.0). The second set of experiments focused on interpopulational genomic comparisons of *O. bicirrhosum*. The gDNA of *O. bicirrhosum* from TAb was compared with the gDNA of *O. bicirrhosum* from AMb against metaphase chromosomes of individuals from TAb. For this purpose, the gDNA of individuals from TAb was labeled with Aminoallyl-dUTP - ATTO-550 (red), while the gDNA of individuals from AMb was labeled with Aminoallyl-dUTP - ATTO-488 (green), both using Nick-translation labeling kit (Jena Bioscience, Jena, Germany). The final probe cocktail was composed by 500 ng of gDNA of individuals from TAb + 500 ng of gDNA of individuals from AMb + 15 μg of derived C0t-1 DNA of specimens from both populations. The probes were ethanol-precipitated, and the dry pellets were dissolved in hybridization buffer containing 50% formamide, 2 × SSC, 10% SDS, 10% dextran sulfate and Denhardt´s buffer, pH 7.0. The CGH experiments were performed according to Symonová et al. [36].

### 2.3. Chromosomal Analyses and Image Processing

At least 30 metaphase spreads per individual were analyzed to confirm the diploid chromosome number (2n) and CGH results. The images were captured using an Olympus BX50 microscope (Olympus Corporation, Ishikawa, Japan) with Cool SNAP, and processed using Image-Pro Plus 4.1 software (Media Cybernetics, Silver Spring, MD, USA).

### 2.4. DArTseq Procedure

Tissue samples (Table 1) were sent to Diversity Arrays Technology Pty Ltd., Canberra, Australia for obtaining genotypic information from SNPs isolated by DArTseq sequencing technique. Genomic DNA complexity reduction in DArTseq is achieved using a combination of a restriction enzymes SbfI and PstI, an enzyme sensitive to some types of methylation, causing the exclusion of methylated and repetitive DNA fragments from the process [37,38]. Therefore, genomic libraries prepared under this method tends to enrich for sequences located in functional genic regions. Resulting libraries were sequenced on an Illumina HiSeq2500 platform.

### 2.5. DArT Data Filtering

**Figure 2:**
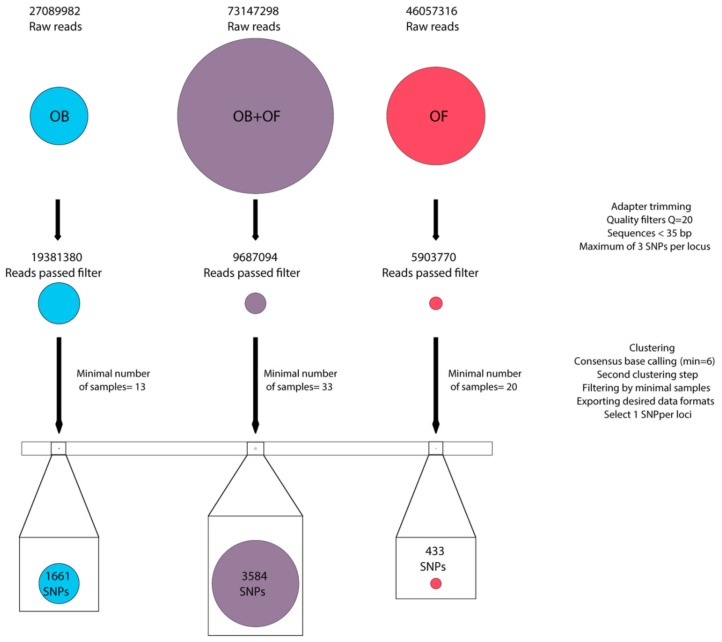
Graphical representation of the 3 analyzed datasets. Circles on the top represent the total amount of raw reads. After filtering steps, the circles sizes show the proportion of reads that passed each filtering step. The circles at the bottom represent the proportion of SNPs in the final matrix, zoomed in. OB = *Osteoglossum bicirrhosum*; OF = *Osteoglossum ferreirai*.

Demultiplexed raw data, provided by the sequencing facility, was processed with ipyrad v.0.7.28 [39]. Sequencing adapters were trimmed, and all sequences with more than 5 low-quality bases (Q < 20) or shorter than 35 base pairs were discarded. The maximum number of SNPs allowed per locus was set to 3 and maximum uncertain base pairs accepted per cluster was defined as 5. The data was then de novo clustered and aligned. Specifically, high-quality consensus sequences were detected for each sample by aligning their reads separately and considering only clusters with more than 6 reads as a pre-locus. After that, the obtained pre-loci were clustered among individuals and only clusters observed in all samples were retained as definitive loci (Figure 2). This last step was carried in three different datasets, one of them comprising both species (combined dataset), one for *O. ferreirai* only (*O. ferreirai* dataset) and the other for *O. bicirrhosum* only (*O. bicirrhosum* dataset). This approach was adopted to maximize the number of markers for subsequent analyzes. Only one aleatory SNP per locus was maintained from the selected loci.

### 2.6. Detection of Outlier Markers Putatively under Selection

In order to search for markers that are possibly under selection, a BayeScan [40] analysis was carried out for the combined and the *O. bicirrhosum* datasets. Runs were executed with a prior odd value of 100, 5000 MCMC chains, and thinning of 10. A total of 20 pilot runs of 5000 iterations were done, burn-in was set to 50,000. Loci with a False Discovery Rate (FDR) value lower than 0.01 were considered as outliers.

### 2.7. Genetic Diversity

Genetic diversity was assessed with Genodive [41]; summary statistics (*H*_O_—observed heterozygosity; *H*_E_—expected heterozygosity; *G*_IS_—inbreeding coefficient) were generated for *O. bicirrhosum* and *O. ferreirai* separately. Pairwise *F_ST_* between all *O. bicirrhosum* sampling locations was also recovered.

### 2.8. Interspecific Differences Structure

The genetic diversity distribution between the two species was assessed with a principal coordinate (PCoA) analysis carried in the R package dartR [42]. A subsequent PCoA was used to visualize the genetic diversity distribution of *O. bicirrhosum* across the two basins, using the *O. bicirrhosum* dataset.

Population structure was also assessed using the Bayesian approach implemented in fastSTRUCTURE v.1.0 [43], a method based on the Bayesian software STRUCTURE [44] but optimized for larger sets of data. In order to prepare inputs and run fastSTRUCTURE, the “Lizards-are-awesome” pipeline [45] was used. The combined dataset was tested with K (number of genetic clusters) ranging from 1 to 5, *O. ferreirai* dataset with K from 1 to 2, and *O. bicirrhosum* dataset with K from 1 to 4.

Because of the small number of localities, isolation by distance (IBD) tests would not have statistical power to give reliable results. Therefore, the spatial analysis implemented in GENELAND [46] was also used to assess population structure only in the *O. bicirrhosum* dataset, as it contains multiple localities. Spatial methods are usually less prone to artificial cluster detection in the presence of isolation by distance (IBD) and are advised for studies with focal groups more prone to IBD, as freshwater fishes [47]. The runs were carried out with a range of K from 1 to 4, 500,000 iterations and a thinning of 200. The correlated allele frequency model was set, and a total of 10 independent runs were performed. The results of both Geneland and fastSTRUCTURE were zipped and uploaded to Structure Harvester [48], for preparation of CLUMPP [49] input files. Graphical outputs were then generated in Clumpak [50].

### 2.9. Demographic Model Selection

To compare demographic models in *O. bicirrhosum*, genetic data were simulated in ms [51] based on our SNP data sample sizes. Priors were selected based on a generation time (G) ranging from 1 to 2 years, according to Queiroz and Camargo [9]. A mutation rate (μ) of 1.25 × 10^−9^ mutations per site per year was used, according to suggested by Oliveira et al. [52] for the genus *Arapaima* (Osteoglossiformes, Osteoglossidae). Effective population size (*N*_e_) was sampled from a uniform distribution ranging from 10,000 to 1,000,000 individuals. Four different scenarios were selected to simulate 50,000 datasets in each of them with a Python script modified from Perez et al. [53]. The first scenario (model 1) consisted of a panmictic population, the second scenario (model 2) comprised a vicariant event with a division in two populations of the same size, the third scenario (model 3) considered a colonization event from AMb to TAb, and the fourth one (model 4) simulated the opposite event, a colonization from TAb to AMb basin. For each simulation, a value of θ was calculated based on the 4*N_e_* μ formula. The divergence time between the basins (DT) was set to 0 on model 1 and sampled from a uniform distribution between 0 and 2 million years ago (MYA) for the subsequent models. The coalescent divergence time (CT), which is the required parameter in ms, was calculated from each sampled DT value based on the DT/4*N_e_*G formula. The intensity of the population reduction during colonization (θrF-A) was estimated as a ratio of the θ value during colonization over the θ in the ancient population. Values were sampled from a uniform distribution ranging from 0.01 to 0.1. For the magnitude of population expansion after colonization (θrC-A), the ratio between θ values in the contemporary and in the ancient population (sampled from 0.1 to 1) were used. Only models 3 and 4 considered the priors for founder effect intensity and growth ratio.

The simulated scenarios were then compared with a convolutional neural network (CNN). CNN is an artificial intelligence method that can be used to learn to identify patterns and then predict those patterns on new data, not seen before by the network. The architecture of the CNN consists of a set of layers that apply mathematical functions to the data that passes through it, generating outputs for subsequent layers. Convolutional layers are followed by pooling layers that summarize the results from previous layers. CNNs can be applied to genetic data because of its high capacity to detect patterns on images and matrices [54]. Our genetic data, organized on matrices of presence or absence of alleles, were converted to images and explored with the approach described by Flagel et al. [54]. We used the same architecture proposed by Oliveira et al. [52], modified from Flagel et al. [54]. The input for the CNN contained 50,000 and 2000 simulations as train and test data, respectively. The run was performed for 25 epochs, with a mini-batch size of 250. After selecting the most likely model, a dataset of 10^6^ simulations was generated under this model, for further parameter estimation using the same neural network architecture. To infer the estimation capacity of the CNN, a Root Mean Squared Error (RMSE) and Spearman’s *ρ* were calculated for each parameter

### 2.10. Paleogeographic Modeling

Potential climatic niche for *O. bicirrhosum* and *O. ferreirai* were estimated for both species based on occurrence data for *O. bicirrhosum* (3 localities from the current study; 3 localities from Verba et al. [55]; 54 localities from Species Link database, http://splink.cria.org.br; and 89 localities from the Global Biodiversity Information Facility—GBIF, https://www.gbif.org/) and *O. ferreirai* (one locality from the current study; six localities from Queiroz and Camargo [9]; two localities from Species Link database; and 16 localities from GBIF). Models were constructed in biomod2 [56] with combination of nine distribution algorithms, including *artificial neural networks* (ANN; [57]), *mixture discriminant analysis* (MDA; [58]), *multivariate adaptive regression splines* (MARS; [59]), *surface range envelop* (SRE; [60]), *classification tree analysis* (CTA; [61]), *generalized linear models* (GLM; [62]), *generalized boosted models* (GBM; [63]), *random forests* [64], and *maximum entropy* (Maxent; [65]). Calibration of the models was performed with present climatic conditions at 30 arc-seconds resolution. Projections for the present, last glacial maximum (21,000 years ago) and last interglacial (120,000 years ago) were carried out at 2.5′ arc-minutes resolution. A total of 5 simulations were performed with each algorithm and only simulations with True Skill Statistic (TSS) higher than 0.7 were kept. Correlation among 15 bioclimatic variables from WorldClim [66] was checked with Pearson index. For variables with high correlation (Pearson index >0.85), only the variable with higher explanatory capacity was maintained.

## 3. Results

### 3.1. Cytogenetic Data

**Figure 3:**
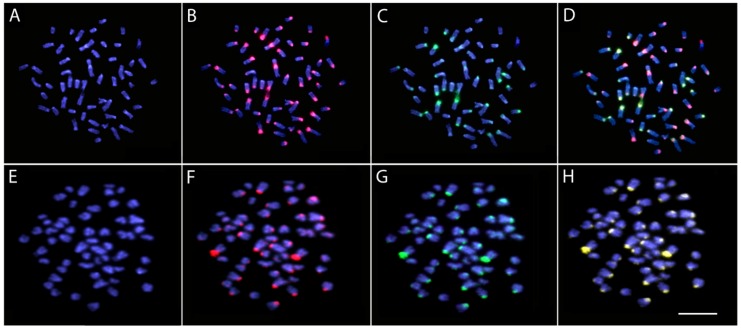
Comparative genomic hybridization (CGH) for intra- and interspecific comparison in chromosomes of *Osteoglossum ferreirai* (**A**–**D**) and *Osteoglossum bicirrhosum* (AMb population) (**E**–**H**). Genomic probes from both *O. ferreirai* and *O. bicirrhosum* hybridized together on background chromosomes of *O. ferreirai* (**A**–**D**) and genomic probes from *O. bicirrhosum* (TAb population) and (AMb population) hybridized together onto background chromosomes of *O. bicirrhosum* (TAb population) (**E**–**H**). First column (**A**,**E**): DAPI images (blue); second column (**B**,**F**): hybridization pattern using gDNA of *O. ferreirai* (**B**) and gDNA of *O. bicirrhosum* (TAb population) probes (red); third column (**C**,**G**): hybridization pattern using gDNA of *O. bicirrhosum* (AMb population) probes (green); fourth column (**D**,**H**): merged images of both genomic probes and DAPI staining. The common genomic regions are depicted in yellow. Bar = 5 µm.

While the silver arowana (*O. bicirrhosum*) has 2n = 56 for all populations analyzed, the black arowana (*O. ferreirai*) differs by presenting 2n = 54 as previously described in Suzuki et al. [67]. In the cross-species hybridization, the gDNA probes from both *Osteoglossum* species showed a high diversity level emphasizing their repetitive DNA content. In fact, only a limited number of overlapping hybridization signals were observed, restricted to the heterochromatic blocks, which were widely present in the centromeric regions of some chromosomes. Several chromosome pairs of *O. ferreirai* showed species-specific sequences with abundant hybridization signals on their centromeric region (Figure 3a–d). The comparative hybridization of the gDNA probes between *O. bicirrhosum* from Tocantins-Araguaia and Amazon localities produced basically a large number of overlapping hybridization signals, highlighting the heterochromatic blocks that occur in the centromeric and terminal regions of some chromosomes. No substantial genomic differentiation in their repetitive DNA content was observed (Figure 3e–h).

### 3.2. Sequencing and Filtering

The DArTseq procedure resulted in a total of 73,147,298 raw reads obtained for all specimens of *Osteoglossum*. From these total reads, the combined dataset resulted in 9,687,094 reads after applying the quality filters presented in methods. After clustering and subsequent filtering, 3584 polymorphic SNP markers were obtained, with 0.38% of missing data. The *O. ferreirai* dataset included 46,057,316 raw reads and, after trimming and filtering, only 5,903,770 reads remained. A total of 433 bi-allelic markers passed the last filtering step with 3.77% of missing data. The *O. bicirrhosum* dataset included 27,089,982 raw reads, from which 19,381,380 reads passed first filtering, and resulted in a total of 1661 SNPs retained at the end of the process with 0.95% of missing data. Though the ipyrad was set to recover only loci available in all samples in each of the three datasets, the amount of missing data reported refers to multiple SNPs located at the same loci. A scheme of the number of retained reads for each step in the three analyzed datasets is presented in Figure 2.

### 3.3. Detection of Markers Putatively under Selection

For both tested datasets, no locus was selected as a candidate under selection. All FDR values were above 0.95 on the combined dataset and higher than 0.97 for *O. bicirrhosum* dataset. Based on this result, no marker was removed in further analyses.

### 3.4. Genetic Diversity

Higher genetic diversity was observed in *O. bicirrhossum* from AMb for all three diversity indexes (A, H_E_, and H_O_) even with fewer samples (Table 2). Expected heterozigosity (H_E_) was slightly higher than the observed (H_O_) for all tested populations. Inbreeding levels were smaller for Tocantins-Araguaia locations and higher in Amazon locations as suggested by G_IS_ values (Table 2). Pairwise F_ST_ estimates reinforced the similarity of the TAb specimens (0.059), with a high differentiation from samples of AMb (0.504 and 0.475 when Catalão was compared with Luciara and Loroti, respectively). The individuals of *O. ferreirai* presented an H_E_ value higher than that of the individuals of *O. bicirhossum* from TAb but lower than that of the individuals of *O. bicirhossum* in AMb. A higher G_IS_ was also observed in *O. ferreirai* when compared to all other localities, suggesting higher levels of inbreeding.

### 3.5. Population Structure

**Figure 4:**
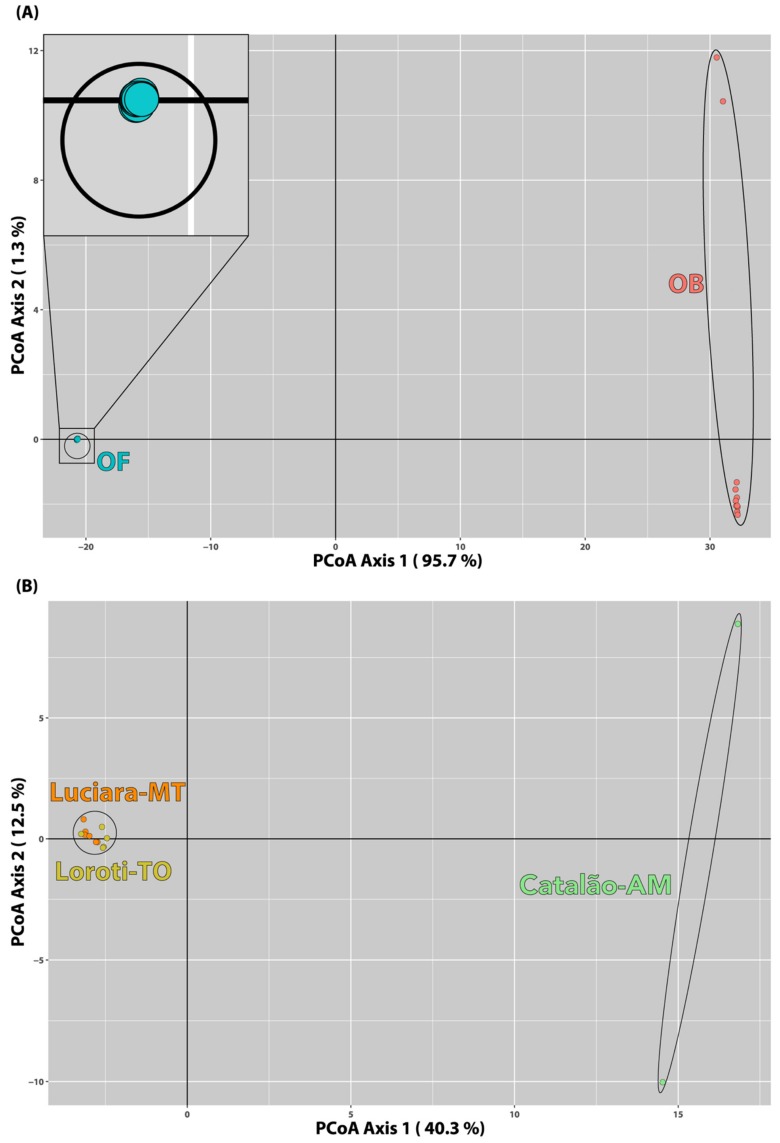
(**A**) Principal coordinate analysis of *O. ferreirai* (blue) and *O. bicirrhosum* samples (red). Insert shows a zoom in *O. ferrerai* samples. (**B**) Principal coordinate analysis of *O. bicirrhosum* samples, with AMb samples (green) and both TAb localities, Luciara (orange) and Loroti (yellow).

The PCoA containing both species of *Osteoglossum* (Figure 4A) showed that specimens from each species are more distantly related than specimens from the two localities of *O. bicirrhosum*. Moreover, *O. ferreirai* had less genetic differentiation among the individuals analyzed, as all of them clustered very close. On the other hand, *O. bicirrhosum* showed a clear differentiation between the individuals sampled in both the same or different basins. The PCoA comprising only *O. bicirrhosum* enhances the difference between the individuals from both basins (Figure 4B).

**Figure 5:**
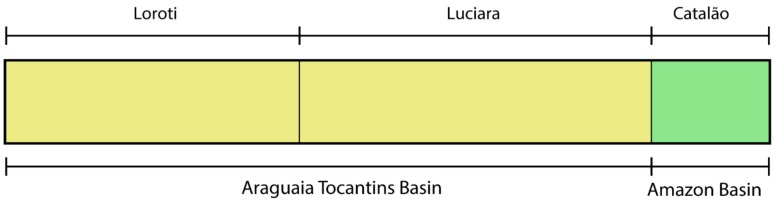
FastStructure and Geneland analysis of *O. bicirrhosum* samples, that had a congruent result returning *K* = 2. Each vertical bar represents an individual and its color represent the proportion of belonging to each population. Black bars separate sampling locations.

Analyses on fastStrucure resulted in a number of clusters that maximizes both likelihood and is more informative for structure with *K* = 2 in the combined dataset, containing all analyzed specimens and separating the two species. The dataset recovered a *K* = 1 for *O. ferrerai* and a *K* = 2 for *O. bicirrhosum*, thus separating samples from the two basins. The GENELAND results of *O. bicirrhosum* dataset were concordant with fastStrucure, returning a *K* = 2, that separates populations based on their basins (Figure 5).

### 3.6. Demographic Model Selection

**Figure 6:**
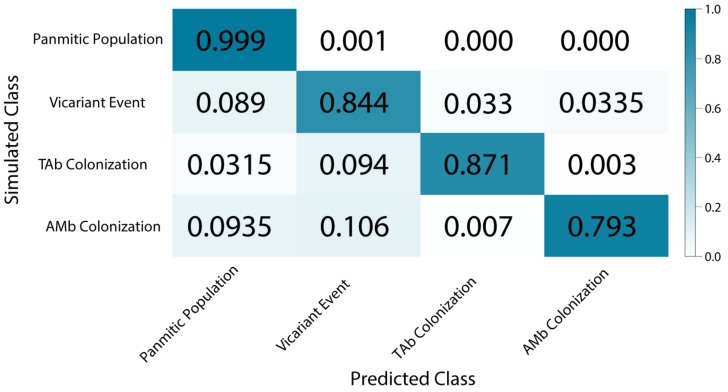
Confusion matrix of deep learning model selection accuracy in predicting correct classes. Diagonal line evidences correct predictions, off-diagonal rectangles, show incorrect predictions. The intensity of blue color and values indicates the accuracy.

During the neural network training process in the *O. bicirrhosum* dataset, the CNN reached an accuracy of 87.5% with the training set, and 87.9% of accuracy in the validation dataset, after 25 epochs. When using only simulated data to predict accuracy, scenario 1 (panmictic) was the easiest to predict with 99% accuracy. The scenario 2 (vicariance) had an accuracy of 84%, while the third (TAb colonization) and the fourth (AMb colonization) scenarios had 87.1% and 79.3% of accuracy, respectively (Figure 6). Accuracy levels higher than 80% are considered acceptable for CNNs [68], therefore our approach presented overall appropriate values. After training and validation, the empirical data were submitted to CNN. The TAb colonization scenario had the highest posterior probability (PP = 0.872), while the panmictic model presented the lowest probability (PP = 0.000).

A parameter estimation step was then carried under the TAb colonization scenario (Table 3). Effective population size (RMSE = 0.4971; Spearman’s *ρ* = 0.6409) and divergence time (RMSE = 0.4816; Spearman’s *ρ* = 0.6571) were the two parameters that CNN had the highest capability to infer. Conversely, founder effect ratio (RMSE = 0.6539; Spearman’s *ρ* = 0.5670) and growth ratio (RMSE = 0.6454; Spearman’s *ρ* = 0.5702) were predicted with less efficiency. Effective population size estimate had a median of 468,195 (interval= 452,302; 481,932). Separation time between basins was estimated at Pleistocene, with a median of 1,049,955 (interval = 1,013,205; 1,074,667). Founder size ratio was estimated with a median of 0.0551 (interval= 0.0538; 0.0561). The estimated growth rate has a median of 0.6454 (interval= 0.5337; 0.5596).

### 3.7. Paleogeographic Modeling

**Figure 7:**
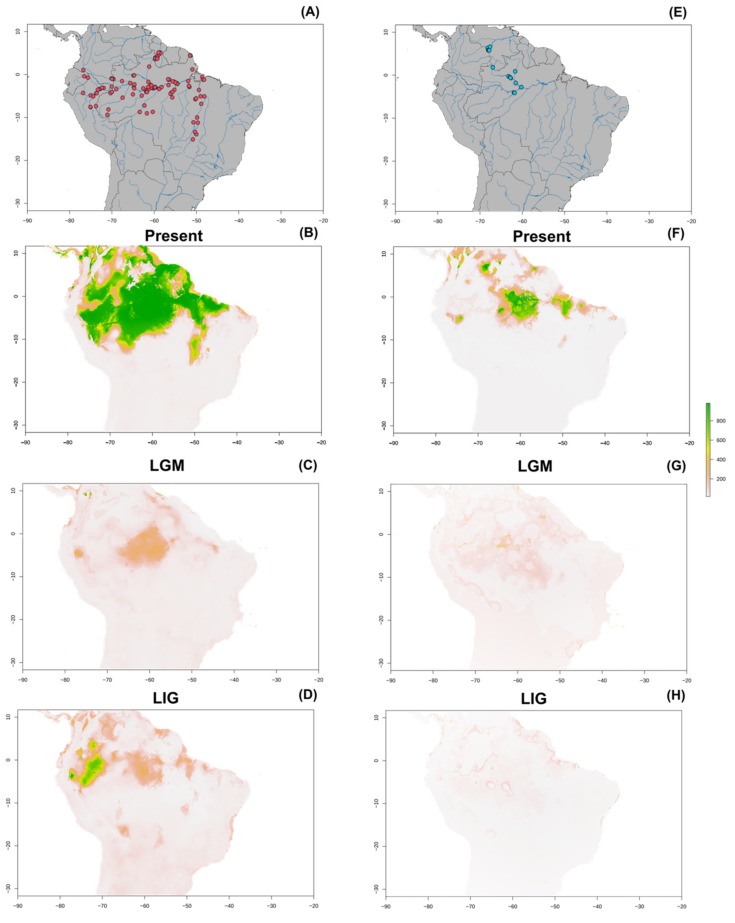
(**A**) Climatic niche modeling in *O. bicirrhosum* according to registered occurrences. Appropriate climatic regions are evidenced by gradient colors for present (**B**), last glacial maximum (**C**) and last interglacial (**D**). (**E**) Climatic niche modeling in *O. ferreirai* according to registered occurrences. Appropriate climatic regions are evidenced by gradient colors for present (**F**), last glacial maximum (**G**) and last interglacial (**H**). Colors refer to environmental suitability, with green representing high and orange low to moderate suitability.

Based on the explanatory capacity and correlation between variables, only seven bioclimatic variables were selected (namely 1, 2, 3, 4, 14, 15, 16) for *O. bicirrhosum*. The analysis generated a distribution in the present that is highly concordant with registered occurrences (Figure 7A,B). The present projection had the broadest distribution among the three simulated periods (Figure 7B). On the last glacial maximum (LGM) the distribution is far more restrictive, only with a few marginally suitable areas, located predominantly in Amazon and some other regions distributed across the north of South America (Figure 7C). The last interglacial period presented a stable area at the current Peru region and other lesser stable locations among Amazon and northern regions of South America (Figure 7D). For *O. ferreirai*, five bioclimatic variables were maintained (3, 7, 11, 12, 14). The present distribution was also congruent with the occurrences (Figure 7E,F), though some areas with clear water were recovered, for example a stable location near Marajó island, more to the east than the current known distribution. The last glacial maximum simulation showed few marginally stable areas, mainly at central Amazon (Figure 7G). During the last interglacial (LIG), there were even fewer suitable areas, presenting only small regions in Amazon and Colombia (Figure 7H).

## 4. Discussion

The two analyzed species have different 2n: the silver arowana, *O. bicirrhosum*, 2n = 56, and the black arowana, *O. ferreirai*, 2n = 54. These results corroborate their distinct species status and thus indicate that they already substantially diverge from one another. Accordingly, these species are also associated to different water characteristics in the environments they live (i.e., different habitat preference) [69,70], and there is no evidence of interspecific gene flow between them at the few sympatric sites (e.g., Demeni River) [9]. In addition, their genomic comparison also showed that both species differ in the composition and distribution of repetitive sequences (Figure 3a–d). A similar scenario is also found in other species of Osteoglossiformes belonging to the family Notopteridae, where most species differ by a significant genomic diversity highlighted by CGH and DArT-Seq analysis [25,71].

Overall, the genetic diversity of *O. bicirrhosum* from the AMb is higher than *O. ferreirai*. DaSilva [69] also evidenced that *O. bicirrhosum* has higher diversity levels analyzing microsatellite markers. This can be related to the restricted distribution and the conservation status of *O. ferreirai*. Our results suggest that interference of past climatic and demographic events, along with contemporary habitat degradation might be factors that influenced this loss of diversity. In addition, economic features may have had also some additional implication. It is known, for example, that when *Scleropages formosus* fishing was restricted in the 1970s [72] not only the fishery on *Osteoglossum* increased, but *O. ferreirai* reached higher selling prices compared to *O. bicirrhosum* [9].

No substantial differentiation in the repetitive DNA content of the distinct *O. bicirrhosum* populations was observed by CGH experiments (Figure 3e–h). Notably, this low evolutionary differentiation at the chromosomal level does not match that of the SNPs dataset. SNPs analyses of population structure highlighted two different populations for *O. bicirrhosum*, one in the AMb and other at the TAb basin (Figure 5). The genetic distance between *O. bicirrhosum* and *O. ferreirai* is larger than intraspecific distances among *O. bicirrhosum* populations (Figure 4). It is important to note that our genetic results should be taken with care, as our sampling sizes are very small (i.e., just 13 specimens with only two analyzed in AMb basin, see Table 1) and do not cover the whole distribution of the analyzed species. Though such small sample sizes can impact the diversity estimates and some of the analyses, the high number of molecular markers used might compensate for the limited numbers of specimens [73]. Moreover, the results presented here are congruent to those obtained with DArTseq markers in *Arapaima gigas* [52], a species with a similar distribution to *O. bicirrhosum*.

Based on the current niche projections for both species in the present conditions, we recovered a potential distribution that was similar, but larger than their natural occurrence. Our modeling approach used only bioclimatic variables, an approach similar to other authors when analyzing freshwater fishes (e.g., [74,75,76]). We selected this strategy, without incorporating other potentially important information, as the presence of floodplains, as such information would preclude projections for past periods. By comparing silver arowana current and past projections, our paleogeographic modeling results indicated that some periods may have been remarkably harsh for these fishes. During the last interglacial period (LIG), that occurred ~120,000–140,000 years ago, *O. bicirrhosum* may have had smaller populations restricted to AMb and close areas. When considering the suitable niche on the last glacial maximum (LGM), at ~21,000 years ago, the distribution is even more restricted. At that time, the Amazon region had several suitable areas that could have served as a shelter for *O. bicirrhosum* populations. Otherwise, the TAb region had a very small area that could have supported such populations. Climatic niche modeling in *O. ferreirai* evidenced LIG as the harsher period, with the smallest projected suitable area. In such conditions, the species may have suffered a decrease in the population size, with the presence of small populations in Colombia and, maybe, in other Brazilian regions. The simulation suggests that during the LGM, *O. ferreirai* experienced more marginally suitable regions, mainly in central Amazon basin, where some populations may have persisted. Comparisons between species indicate that *O. ferreirai* niche was smaller at all periods, which could be related to the habitat preference of this species for dark acid waters. The population reduction in both species should have concomitantly decreased their genetic diversity due to genetic drift. Besides such factors, the hydrological dynamics that occur in the TAb may also directly impact the species diversity levels. This is the case of the flooding events that connect some rivers allowing gene flow and thus making populations more homogenous. In addition, the species behavior cannot be omitted either, as *O. bicirrhosum* is characterized as a non-migratory species [22], that tends to form local communities leading to higher inbreeding levels.

Our deep learning approach for model comparison selected a colonization scenario from AMb to TAb. The parameter estimation step indicated that TAb colonization occurred during the Pleistocene (median = 1,049,955.4 years ago), and this result agrees with the suggestion of Rossetti and Valeriano [77] about the age of separation for the TAb and AMb. This result can also be interpreted as TAb having a smaller population before the separation. The effective population size recovered had a median of 468,195, four times greater than the values recovered for *Arapaima* species [52], another species of the family Osteoglossidae with a similar distribution. This difference may be related to the biological characteristics of both species: *A. gigas* is a very large species, while *O. bicirrhosum* is much smaller and usually much more abundant where both species occur in sympatry (E.A.O. personal observation). The other parameters estimated showed low Spearman’s *ρ* and higher RMSE, evidencing lower capacity of CNN to predict these values. It is important to note that several results obtained here are congruent with recent populational assessments of *A. gigas* [52,78,79], comprising higher genetic diversity in Amazon populations compared to those of TAb, and a scenario of TAb colonization from an ancient Amazon population in a similar timeframe (~900 kya, [52]). This congruence indicates a similar evolutionary history for both *O. bicirrhosum* and *A. gigas*, mediated by the separation of the TAb and AMb, which can be also responsible for promoting genetic structuring and differentiation in other fish species. Otherwise, distinct events might have promoted the divergence between *O. ferreirai* and *O. bicirrhosum*, as both species occur in the Amazon basin, mostly in allopatry, and estimated divergence age is much older. In this sense, as indicated previously, water characteristics associated with different chemical properties may be related to ecological speciation in fishes as found in some other freshwater fishes (e.g., [70]).

## 5. Conclusions

In summary, our results provided evidence of an overall higher genetic diversity in arowanas from the Amazon basin compared to those from the TAb, suggesting that the latter may be more likely to reach a vulnerable conservation status. It also pointed out that populations from the same basin are more similar genetically and that the species divergence is more ancient than the separation of populations in different basins. For both black and silver arowanas, paleomodeling suggested a larger distribution nowadays than in the past, and our demographic model approach pointed to the colonization of the TAb from an ancient Amazon population in *O. bicirrhosum* during Pleistocene. When comparing both species, *O. ferreirai* probably requires a more elevated conservation status than that of *O. bicirrhosum*, because of its restricted distribution and its association to a specific water type.

## Figures and Tables

**Table 1:** Collection sites of the arowana species analyzed, with the corresponding numbers of individuals used for single nucleotide polymorphisms (SNP - DArT_N) and cytogenetic analyses (Cito_N).

Species	Sampling Site	River Basin	DArT_N	Cito_N
*Osteoglossum bicirrhosum*	Javaé River (Loroti, TO)	Tocantins-Araguaia	5	(06♀ 08 ♂)
*Osteoglossum bicirrhosum*	Xavantinho River (Luciara, MT)	Tocantins-Araguaia	6	(06♀ 05 ♂)
*Osteoglossum bicirrhosum*	Solimões River (Catalão, AM)	Amazon	2	(10♀ 09♂)
*Osteoglossum ferreirai*	Negro River (Tarumã, AM)	Amazon	20	(11♀ 10 ♂)

**Table 2:** Genetic diversity levels of *Osteoglossum* species. A—average number of alleles; *H*_O_—observed heterozygosity; *H*_E_—expected heterozygosity; *G*_IS_—inbreeding coefficient.

Locality	Species	Basin	A	*H*_O_	*H*_E_	*G*_IS_
Luciara	*O. bicirrhosum*	TAb	1.202	0.121	0.137	0.117
Loroti	*O. bicirrhosum*	TAb	1.211	0.128	0.145	0.123
Catalão	*O. bicirrhosum*	AMb	1.433	0.275	0.358	0.232
Tarumã	*O. ferreirai*	AMb	1.235	0.112	0.176	0.364

**Table 3:** Demography parameter estimates of *Osteoglossum bicirrhosum* based on the selected demographic model. *N*_e_—Effective population size; DT—divergence time between the two basins; *θ*rF-A—Population size reduction ratio during colonization; *θ*rC-A—the ratio between the current and the ancient population.

Parameter	RMSE	Spearman’s *ρ*	Median	Interval
*N*_e_	0.4971	0.6409	468,195.3679	452,302.7–481,932.6
DT	0.4816	0.6571	1,049,955.4491	1,013,20–1,074,667.3
*θ*rF-A	0.6539	0.5670	0.0551	0.0538–0.0561
*θ*rC-A	0.6454	0.5702	0.5470	0.5337–0.5596
